# Nerve Growth Factor mRNA Expression in the Regenerating Antler Tip of Red Deer (*Cervus elaphus*)

**DOI:** 10.1371/journal.pone.0000148

**Published:** 2007-01-10

**Authors:** Chunyi Li, Jo-Ann L. Stanton, Tracy M. Robertson, James M. Suttie, Philip W. Sheard, A. John Harris, Dawn E. Clark

**Affiliations:** 1 AgResearch, Invermay Agricultural Centre, Mosgiel, New Zealand; 2 Department of Anatomy and Structural Biology, University of Otago, Dunedin, New Zealand; 3 Department of Physiology, University of Otago, Dunedin, New Zealand; Centre de Regulacio Genomica - Barcelona Biomedical Research Park, Spain

## Abstract

Deer antlers are the only mammalian organs that can fully regenerate each year. During their growth phase, antlers of red deer extend at a rate of approximately 10 mm/day, a growth rate matched by the antler nerves. It was demonstrated in a previous study that extracts from deer velvet antler can promote neurite outgrowth from neural explants, suggesting a possible role for Nerve Growth Factor (NGF) in antler innervation. Here we showed using the techniques of Northern blot analysis, denervation, immunohistochemistry and in situ hybridization that NGF mRNA was expressed in the regenerating antler, principally in the smooth muscle of the arteries and arterioles of the growing antler tip. Regenerating axons followed the route of the major blood vessels, located at the interface between the dermis and the reserve mesenchyme of the antler. Denervation experiments suggested a causal relationship exists between NGF mRNA expression in arterial smooth muscle and sensory axons in the antler tip. We hypothesize that NGF expressed in the smooth muscle of the arteries and arterioles promotes and maintains antler angiogenesis and this role positions NGF ahead of axons during antler growth. As a result, NGF can serve a second role, attracting sensory axons into the antler, and thus it can provide a guidance cue to define the nerve track. This would explain the phenomenon whereby re-innervation of the regenerating antler follows vascular ingrowth. The annual growth of deer antler presents a unique opportunity to better understand the factors involved in rapid nerve regeneration.

## Introduction

Nerve Growth Factor (NGF) is involved in many aspects of nerve growth. This small secreted dimeric protein, originally identified by its ability to promote neurite out-growth, exerts its biological actions primarily on cells of the nervous system [1]. Binding of NGF to p75 and trk family receptors on responsive neurons has been shown to result in their survival and growth during both embryogenesis and regeneration [2]. NGF is responsible for the survival of sympathetic and sensory neurons concerned with nociception and thermoreception and all autonomic axons are NGF sensitive. Developing peripheral nerves induce expression of NGF in their targets during embryo development while upregulation of NGF expression by Schwann cells following nerve injury is thought to facilitate growth of regenerating peripheral axons back toward their original targets [3]. This subtle difference separates embryonic NGF expression from that during regeneration. It is, therefore, possible to distinguish “generation” in which the nerve induces NGF expression by the target that in turn permits nerve survival, from “regeneration” when the target expresses NGF to attract axon regrowth.

NGF has also been found to have non-neuronal functions [4]. Notably NGF has been implicated in the process of angiogenesis with the trkA^NGFR^ and p75^NTR^ receptors for NGF present on Human Umbilical Vein Endothelial Cells [5]. Other significant functions of NGF have been in association with tissue repair and the differentiation associated with ovarian development and folliculogenesis [6].

NGF is synthesized as a prohormone of 22 kDa which undergoes proteolytic cleavage. The three dimensional structure of NGF is determined by the presence of six cysteine residues involved in disulphide bond formation. These are important in defining the backbone structure of the protein [7]. The gene for NGF consists of several small exons coding for multiple precursor forms with the nerve growth promoting activity confined to exon IV [8].

Deer antlers are elaborate bony structures that are shed and then completely regenerate each year. This cycle of antler casting and regeneration is controlled by seasonal fluctuations of testosterone with antler growth beginning in early spring when testosterone decreases below threshold levels [9]. During their growth phase, antlers are enveloped in a unique type of delicate skin known as “velvet”. Antlers are extremely sensitive to nociceptive and discrete touch stimuli and deer have an awareness of antler position [10]. Velvet antlers are innervated by both unmyelinated and myelinated sensory nerve fibers, which are derived from the supraoptic and temporal branches of the trigeminal nerve, and these nerve fibers follow the same paths as the major arteries [11]. At the end of summer, an increase in circulating testosterone levels causes antlers to become fully calcified and velvet skin to shed. Bony antlers are carried by a stag throughout the winter season and cast in the following spring.

Antler is a valuable model for the study of nerve growth and regeneration as the repetitive renewal of a highly innervated, developmentally regulated organ is not observed in other mammals. Very little is known, however, about nerve regeneration and antler innervation during antler renewal. A previous study from our group [12] revealed that neurotrophin-3 (NT-3), a nerve trophic factor, is widely expressed in the growing antlers. NT-3 expression levels are closely correlated with the density and pattern of innervation of different antler tissue types, being highest in the segment immediately subjacent to the tip and lowest in the cartilage layer. NT-3 is not expressed in the tissue positioned ahead of the most advanced nerve endings. This led the authors to suggest that NT-3 does not play a role in attracting nerve fibers towards their target field, but is only involved in their differentiation and survival. As extracts from velvet antler are able to promote neurite outgrowth from neural explants [13], they must contain other factors to fulfill the role of attracting nerves. Based on current knowledge, NGF appears to be a likely candidate.

In the present study, we showed that NGF mRNA was expressed in the arterial smooth muscle of regenerating antlers. Using these data we proposed a model of antler innervation whereby axons grow into the regenerating antler using NGF expressed by the vascular smooth muscle as a guidance cue to define the nerve track. Full or partial denervation of the regenerating antlers significantly increased NGF mRNA expression in the antler arterial smooth muscle, indicative of a causal relationship between nerve ingrowth and NGF expression. Our model explains the phenomenon whereby reinnervation of the regenerating antler follows vascular ingrowth.

## Results

### Sequencing of Deer NGF

Prior to commencing this work the sequence for deer NGF was unknown. Partial sequence coding for the mature protein was obtained from deer genomic DNA using PCR primers designed from exon IV of the bovine NGF gene. This deer sequence has been deposited in GenBank (Accession number AF145043). We obtained this sequence by analyzing multiple clones (7) from four individual animals. Deer NGF showed 97%, 92% and 88% homology to bovine, human and mouse NGF sequences respectively [14], [15]. Residues shown to be essential for the biological activity of NGF in other species were strictly conserved in the deer sequence. The deduced amino acid sequence for deer NGF showed that the cysteine (nucleotides 172–174, 202–204 238–240), histidine (nucleotides 223–225, 250–252), and tryptophan residues (nucleotides 61–63, 226–228, 295–297) [7] were present. Based on the high level of homology between this sequence and the nucleotide sequence of NGF in other species we concluded that we had isolated the deer NGF homologue.

### NGF mRNA expression in the growing antler tip

**Figure 1:**
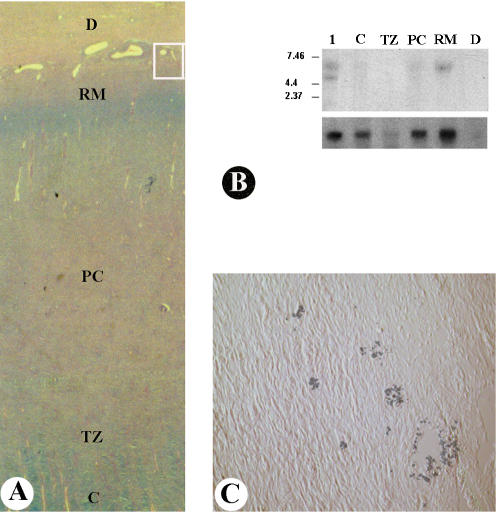
**A.** Layer identification of proliferative zone on a haematoxylin and eosin, and alcian blue stained growing antler tip section. D, dermis; RM, reserve mesenchyme; PC, precartilage; TZ, transitional zone; C, cartilage. Rectangular box indicates position where tissue was sampled for immunohistochemical localization (Figure 1C). **B.** Northern blot analysis showing the distribution of NGF (top) and ß-actin (bottom) mRNA in the antler tip. Blots containing 5 µg of poly(A) RNA from each tissue were hybridized with a 32P-labeled DNA probe. Lane 1) E17.5 mouse embryo; Lane 2) C; Lane 3) TZ; Lane 4) PC; Lane 5) RM; Lane 7) D. The positions of RNA size markers (in kb) are indicated *(left)*. **C.** Immunohistochemical localization on a section of antler tip showing bundles of axons. Serial wax-embedded-sections were labeled with a fluorescent antibody against neurofilament. Fluorescent images have been overlaid onto corresponding bright field view. Axon bundles were not visible when primary antibody was omitted (original size, 10× magnification of original figure).

The expression of NGF in the antler tip was investigated using Northern blot analysis. Northern blots consisting of mRNA from different layers within the antler tip (Fig. 1A) and from a mouse E17.5 embryo were hybridized with a DNA probe for deer NGF. Localized signal corresponding to an NGF transcript of an unexpected size (6 kb) was present in the antler reserve mesenchymal layer (Fig. 1B). This transcript was also present in the mouse embryo sample. Expression of NGF was low when compared with autoradiographic signal obtained from β-Actin. Northern blot analysis was repeated three times using antler tips from three individual deer.

### Immunohistochemical localization of axons in the antler

Earlier studies provided information describing nerves in the shaft of the antler but did not give a clear indication of where axons terminated distally [11], [12], [16], [17]. We wished to determine the positional relationship between NGF expression and axon terminals. Neurofilament immunopositive profiles labeled with rhodamine were detected in antler tissue sections and the consistent appearance of labeled structures with a similar shape, size and position in adjacent longitudinal sections indicated binding of the antibody was specific and reproducible (Fig. 1C). We concluded that these represented axons, based on the known specificity of the antibody and the size and shape of the transverse sectional profiles. Bright field images of the same sections revealed structures resembling bundles of axons. All negative controls had low background and no immunopositive profiles.

**Figure 2:**
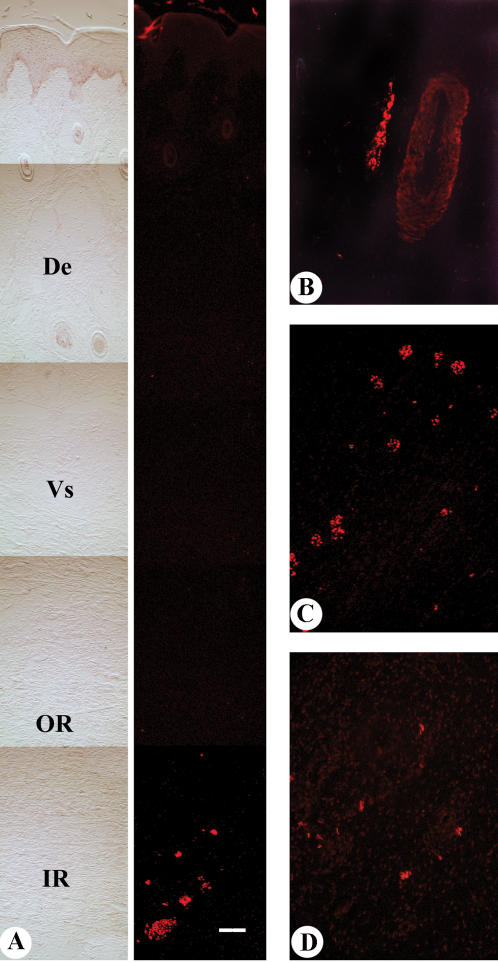
Immunohistochemical localisation of axons on antler tip sections. The same method was used as for Figure 1C. Bar = 100 µm. A. Montage of a longitudinal section of central antler tip showing axon bundles. Bright field is on left. Corresponding fluorescence on the right showing individual axons in red mainly in the inner reserve mesenchyme (IR), not found in dermis (De), vascular layer (Vs) and outer reserve mesenchyme (OR). B. A bundle of axons was near a blood vessel in the side portion of an intact antler tip. C. Numerous fluorescent-labelled neurofilaments were observed in the reserve mesenchymal layer on the sections from the first two denervated antler tips. D. Positive stained neurofilaments were only sparsely encountered in the third denervated antler tip.

Antler tip samples were divided into longitudinal sections from each side of the tip and a central section which spanned from the apex into the core of the antler. Axons with diameters ranging from 0.5–3 µm were found mainly in the reserve mesenchymal layer (Fig. 2A) in the antler tip. Major axons were located near blood vessels growing alongside the mesenchymal layer at the interface with the dermis in the side portion of the antler tip (Fig. 2B). Axons were not detected in the precartilage, transitional or cartilage layers. Axon density varied across the reserve mesenchymal layer with more abundant and larger bundles at the sides of the antler than at the very tip of the antler overlying the core region.

### Axon profiles in denervated antler

Immunohistochemistry was employed to characterize axon patterns in antlers following severing of the zygomaticotemporal and infratrochlear branches of the trigeminal nerve and the auricular palpebral branch of the facial nerve. Contrary to our expectations, 3 weeks after denervation we found the number of immunopositive profiles greatly increased (Fig. 2C) in two of the three denervated antler tips compared to their contralateral controls. Sections taken from the third denervated antler showed only sparse innervation (Fig. 2D) compared to its normally innervated counterpart. Evaluation of the denervation surgery at the completion of the experiment showed that in two of the experimental animals surgery was only partially successful. These corresponded to the first two antlers in which increased axon profiles were observed. There was no apparent growth of axons into the base of the antler belonging to the third animal.

### Cellular localization of NGF mRNA in the developing antler

**Figure 3:**
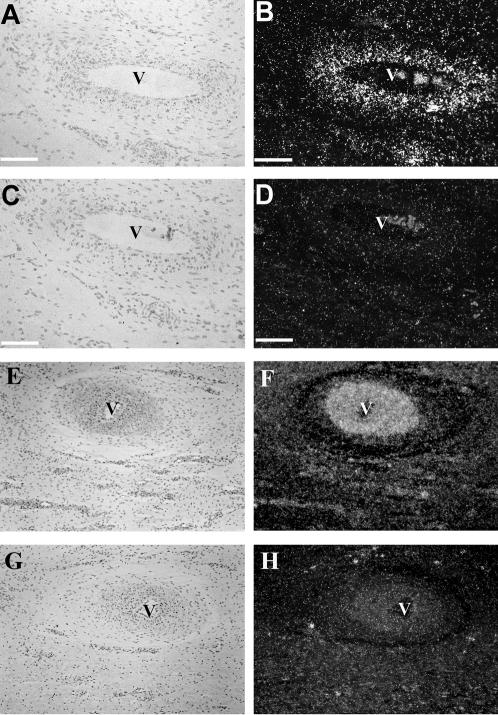
In situ hybridization showing bright (A, C, E, G) and corresponding dark (B, D, F, H) field views of a serial sections through an artery from the intact antler tip (A–D), and an artery from the denervated antler tip (E–H) of the same deer. A and B, E and F binding of the antisense NGF riboprobe, while C and D, and G and H show the sense NGF riboprobe. Bar = 100 um. The magnification of figure 3D applies to the figures 3E to 3H.

To identify cells expressing NGF in the growing antler tip, and to determine whether a feedback mechanism exists between the ingrowing sensory nerve axons and the expression of NGF mRNA, the tips of both denervated and contralateral control antlers were subjected to in situ hybridization using a riboprobe for deer NGF. NGF mRNA was found to be expressed in smooth muscle cells surrounding the arteries and arterioles at the interface between dermis and reserve mesenchyme in the intact antler tip (Fig. 3A–3D), in close proximity to where nerves track in the antler [11], [12], [16]. These NGF expressing cells were located principally in the vascular bed lying at the interface between the reserve mesenchyme and the dermis (layers illustrated in Fig. 1). NGF was expressed in the vascular bed both at the lateral side of the antler and also in the vessels overlaying the most apical point of the tip. This was closer to the antler tip than our localization of the majority of axons. The comparative level of hybridization signal in denervated antler and the contralateral normal control showed an increase in NGF expression following loss of axons (Fig. 3E–3H). The overall pattern of NGF mRNA expression, however, did not change (data not shown). Control (sense) riboprobes gave only scattered background hybridization.

## Discussion

This study has identified nucleotide sequence for deer NGF. PCR and cloning techniques were used to generate a consensus sequence for part of the deer NGF mRNA, and this showed strong homology with NGF of other species. Northern blotting showed an NGF transcript of approximately 6 kb. This message was larger than the expected 1.3 kb size suggested by Scott *et al* [18]. A 3.6 kb transcript for NGF has previously been identified in fish [19] and a larger than expected transcript of a closely related molecule, Brain-Derived Neurotrophic Factor, has been reported in mice [20]. These findings suggest that the 6 kb NGF transcript found in antler, and also in the E17.5 mouse embryo, may code for an NGF precursor initiated from an unidentified exon. Edwards *et al* [21] have proposed that different precursor forms may result in different cellular locations of NGF and could alter the biological activity of the protein.

Localization of NGF mRNA expression was consistent between our Northern blot analysis (Fig. 1B) and in situ hybridization results (Fig. 3). In situ hybridization further demonstrated that the signals were located at the interface between the reserve mesenchyme and the dermal layer of the antler, principally in smooth muscle cells surrounding the arteries and arterioles (Fig. 3). Antler nerves have been shown previously to follow the same tracks as those taken by blood vessels [11], [12], [16]. NGF mRNA expression in these specific cells may therefore delineate the path that axons are to take as they extend commensurate with antler tip growth which, at its peak, reaches a rate of more than 10 mm/day [22]. Conversely, sensory neuron secreted neuropeptides Substance P and Calcitonin Gene-Related Peptide, both of which are found to be highly expressed in the growing antler vascular layer [17], may facilitate blood vessel formation. It is known that both of these neuropeptides play a stimulating role in endothelial cell proliferation [17]. Further, the apparent increase in NGF expression level in these cells following full or partial denervation of the antler suggests that there is a finely balanced feedback system between the entering axon and the newly formed smooth muscle of the antler arterial system.

Comparison of axon density in the distal region with that in the shoulder region of the antler revealed that the shoulder region was more densely innervated than the tip region. The most distal tissues in the antler are those that formed within the last few hours and presumably axons have not yet grown into these newly formed regions. The cellular localization of NGF mRNA suggests that blood vessels at the interface of the reserve mesenchyme and the dermis act either as the peripheral targets or specify the pathway for axons innervating the antler. Gibran *et al* [23] have suggested that microvascular endothelial cells, induced to produce NGF by Substance P, may perform a similar function during healing of cutaneous injury.

The developmental expression of NT-3 correlates well with the density and pattern of innervation in the antler [12]. Buchman and Davies (1993) proposed that neurons pass through a neurotrophin independent phase before they reach their target field, then ‘switch’ to a dependency on neurotrophins for survival. The switch to neurotrophin dependence is thought to be controlled by an intrinsic timing mechanism within the neuron [20]. The observation that regenerating axons extend up the antler shaft and branch to form differentiated sensory terminals, while others continue to extend to their targets further up the antler [16], is consistent with an intrinsic timing program for individual neurons. This would explain why some regenerating axons continue to elongate distally, while others stop to form sensory terminals.

Antler denervation was followed by up regulation of NGF mRNA expression in the antler tip and, in this case, resulted in an increase in the number of axon profiles present near the tip, even when there were few axon profiles remaining at the base of the antler. Surgical axotomy of branches of the trigeminal and facial nerves which provide sensory innervation to antlers is unlikely to have affected autonomic axons which travel with the antler arteries. The over expression of NGF in response to the loss of sensory nerves would stimulate sprouting of parasympathetic axons. Localized over expression of NGF mRNA at the base of the antler following denervation surgery may also explain why two of the experimental animals exhibited an increased number of immunopositive axonal profiles. Alternatively, the high level of NGF mRNA produced in response to denervation may aid the survival of sensory axons in the absence of a cell body. This phenomenon has been observed in the frog by Humphrey *et al* [24], who have suggested that after denervation axons can survive *in vivo* without a cell body for up to three weeks. Elevated NGF levels may maintain sensory axons in a denervated antler, however, the prospect of these axons growing with the antler for a further 10 cm over a three week period is difficult to rationalize.

NGF has also been associated with biological functions other than nerve growth and survival. It has been shown to promote wound healing [25], and have a role in cell differentiation [26], [27]. Its expression can be modulated by retinoic acid [26], [28]. Interestingly Allen *et al* [29] have shown retinoic acid receptors and significant concentrations of Vitamin A and the RALPH2 enzyme distributed throughout the growing antler tip. One could hypothesize that retinoic acid may play a role in regulating NGF expression in the antler vascular bed. In addition, Emanueli *et al* [30] have shown that NGF promotes angiogenesis and arteriogenesis via the vascular endothelial growth factor (VEGF) pathway following ischemic injury, with NGF proceeding VEGF action. A role for NGF in vascularisation, coupled to re-innervation, is further supported by Reimer *et al* [31] who showed enhanced blood vessel and nerve ingrowth to transplanted islet cells in rats treated with NGF or a combination of NGF and VEGF. We have concentrated principally on whether NGF acts as a guidance cue for regenerating antler sensory axons. NGF's presence in the vascular bed of the antler tip, however, may suggest it also forms part of the mechanism for angiogenesis and arteiogenesis in the regenerating antler.

In summary, we have shown that NGF mRNA is expressed in the smooth muscle of the arteries and arterioles in the growing antler tip. Axon pathways in the antler have been shown to follow the major blood vessels supplying this structure. This vascular bed lies at the interface between the dermis and the reserve mesenchyme. The positional relationship between NGF expressed by the blood vessels and the most distal growth of the axons shows NGF mRNA closer to the apex of the antler than the majority of nerve endings. We have also demonstrated that a feedback mechanism exists between NGF mRNA expression in arterial smooth muscle and sensory axons in the antler tip. We therefore propose that axons growing into the antler use NGF expressed by the vascular smooth muscle as a guidance cue to define the nerve track. This means that innervation of the antler follows and is shaped by vascular ingrowth. We suspect that for the sensory axons to form terminals with their sensory target requires a second signal, such as an intrinsic timing mechanism, to complete their development. This model of NGF utilization is most closely aligned to regenerative nerve growth rather than that which occurs during embryonic nerve generation. Therefore, the annual growth of deer antler provides a unique opportunity to better understand the factors involved in rapid nerve regeneration.

## Materials and Methods

### Tissue Collection

**Figure 4:**
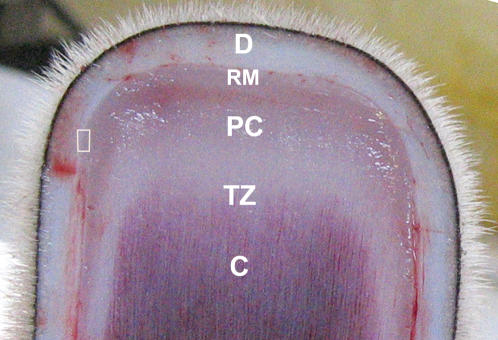
Layer identification of unstained proliferative zone in a growing antler tip. D, RM, PC, TZ, C, are the same as shown in Figure 1. Rectangular box indicates position where tissue was sampled for in situ hybridization.

Velvet antler was collected from red deer (*Cervus elaphus*) 60 days after casting of the previous year's hard antler. Local anaesthetic (bromacaine) was injected around the junction of the pedicle and the skull before each antler was removed above the pedicle. The distal 5 cm from each antler was collected for this study. Following the established methods [32], antler tip tissue for mRNA extraction was rapidly separated into five morphologically defined layers (Fig. 4) under a dissecting microscope, snap frozen in liquid nitrogen and stored at −80°C. These layers were dermis, reserve mesenchyme, precartilage, transition zone, and cartilage. Antler tissue samples for in situ hybridization and immunohistochemistry were immediately fixed in 10% formalin for 24 hours and then transferred to 70% alcohol, prior to wax embedding. All deer were supplied and maintained by the AgResearch Invermay Farm, Mosgiel, New Zealand. The antler removal procedure was conducted in accordance with the regulations set up by the New Zealand National Velveting Standards Board.

### Surgery

Denervation experiments were carried out, following approval by the AgResearch Invermay Animal Ethics Committee, to determine whether there exists a causal relationship between nerve ingrowth and NGF mRNA expression level. Three 2-year-old red deer (*C. elaphus*) stags were selected at 10–20 days post casting of their previous year's antlers. At this stage antlers were between 6 and 8 cm in length. Animals were anaesthetized with Fentazin (1.8 ml/100 kg). The zygomaticotemporal and infratrochlear branches of the trigeminal nerve and the auricular palpebral branch of the facial nerve innervating one of the antlers were cut using the procedure reported by Li *et al* [16], and tied back onto themselves as described by Wislocki and Singer [11]. This resulted in unilateral sensory denervation of the antler. Post surgery, animals were administered 1 ml/10 kg Penstrep LA (BOMAC Laboratories Ltd, Auckland) and their recovery monitored. Deer were maintained outdoors for 19 days at which time their antlers measured between 12 and 17 cm in length. Antlers were removed from the deer and processed as described above. To evaluate the success rate of denervation, all deer were sacrificed at the conclusion of the experiment and exploratory dissections carried out.

### Cloning Procedures and Sequencing

Genomic DNA was extracted from deer whole blood. This material served as the template for PCR using primers designed from exon IV of the bovine NGF gene (Accession number: M26809). The forward primer sequence was 5′-GGGGAGTTCTCGGTGTGCGA-3′ and the reverse primer was 5′-CACACGCAGGCCGTGTCGAT-3′. PCR products were A/T cloned into the pGEM-T® Easy vector (Promega, Madison, WI) containing the SP6 and T7 RNA polymerase promoters flanking the multiple cloning site. DNA was sequenced using primers to the SP6 and T7 promoters and fluorescent dye terminator chemistry on an ABI 377 automated cycle sequencer (Centre for Gene Research, University of Otago).

### Northern Blot Analysis

Isolation of mRNA from antler tissues or whole E17.5 mouse embryos used the MicroPoly(A)Pure mRNA purification kit (Ambion, Austin, Texas). Approximately 5 µg of mRNA was subjected to formaldehyde gel electrophoresis and capillary transferred onto Hybond N+ nylon membrane (Amersham, UK). Twenty five ng of deer NGF DNA was labeled by random priming with 10µCi/µl [α^32^P]dCTP and the probe purified using a NICK™ Column (Pharmacia LKB Biotechnology, Sweden). Hybridization was performed at 42°C overnight in 50% formamide [33]. Autoradiography was performed with an intensifying screen at −80°C.

### Immunohistochemical Localization of Axons

The immunohistochemical protocol for nerve staining followed that described by Li *et al* [16]. Ten µm paraffin antler tissue sections were dewaxed in xylene and rehydrated. The primary antibody raised against a 200 kDa neurofilament polypeptide (mouse anti-pig, Amersham, UK) was diluted 1∶200 in immunodiluent and placed on the sections. Controls for immunohistochemical specificity included substituting the equivalent amount of rabbit immunoglobin (IgG) antibody in place of the primary antibody as well as omission of the primary antibody. The secondary antibody (biotinylated sheep anti-mouse, Amersham, UK) was diluted 1∶200 in immunodiluent and then incubated with the section. This was followed by incubating the sections with a biotin-streptavidin-rhodamine complex. Slides were cover-slipped with gelatin and examined using a Zeiss Axioplan fluorescence microscope. Images were captured using a Pixera professional digital camera.

### In situ Hybridization

The in situ hybridization protocol was based on the methods described by Clark *et al* [34]. A deer NGF PCR product cloned into transcription vector pGEMT® Easy (Promega, Madison, WI) was linearized by restriction digest with either *Sac*II (New England Biolabs, Beverly, MA) or *Sal*I (Boehringer Mannheim, Germany) to give sense and antisense probes respectively. Single stranded sense and antisense riboprobes were labeled by transcription with 10µCi/µl [α^33^P] UTP as per manufacturers instructions (Promega, Madison, WI). Sections were counter stained with Gills Hematoxylin and viewed on an Olympus BX50 microscope using both bright and dark field illumination.
